# Behavior and Practices of Type 2 Diabetic Patients Regarding Obesity: A Cross-Sectional Study

**DOI:** 10.7759/cureus.10126

**Published:** 2020-08-30

**Authors:** Quratulain Akbar, Bilal Ahmed Khan, Bakhtawar Saleem Rajput, Nadia Nazir Jatoi, Sadia Elahi, Abbas Mustafa Gain, Arooba Amjad, Dureshahwar Akbar, Maaz Bin Nazir, Naveed Gianchand

**Affiliations:** 1 Internal Medicine, Dow Medical College, Dow University of Health Sciences, Karachi, PAK

**Keywords:** type 2 diabetes, obesity, diabetes mellitus, diabetes complications, health behavior

## Abstract

Background

Obesity is a major public health concern and is associated with incident cardiovascular diseases. A very few studies around the globe have assessed how type 2 diabetic (T2D) patients comprehend obesity. Our study aims to evaluate the concerns and behaviors of T2D patients regarding obesity in a developing country like Pakistan.

Methods

A cross-sectional study was conducted in Karachi during the period of December to February 2020 in which T2D patients were assessed for their comprehension of how obesity affects their disease and concerns, as well as their practices such as weight loss activities and dietary habits. Data analysis was performed using Statistical Package for the Social Sciences Version 24 (IBM Corp., Armonk, NY, USA).

Results

Of 417 T2D patients inducted in our study, 265 (63.5%) knew their ideal body weight, whereas only 221 (52.9%) knew how to measure it. Among those who were willing to lose weight, this was mostly due to a wish to avoid further complications of obesity (N=155 [73.1%]) and also peer/family pressures (N=124 [58.5%]) among other reasons. More obese (N=68 [43.6%]) than non-obese participants (N=87 [33.3%]) were willing to consult a doctor to help them reduce weight. Participants had adopted various strategies to reduce weight, of which increasing exercise (N=242 [85.8%]) and healthy eating (N=162 [57.4%]) were most popular.

Conclusions

There is a need to address barriers to weight loss among T2D patients in Pakistan and to provide patients with pragmatic guidelines on how to make sustainable lifestyle changes to help reduce and maintain their body weight.

## Introduction

The World Health Organization (WHO) has labeled obesity a global epidemic. Obesity is classified as a body mass index (BMI) of ≥30 kg/m2 and is a major risk factor for insulin resistance leading to type 2 diabetes mellitus (T2DM) [1]. Many epidemiological studies support an association between T2DM and obesity [2,3].

According to the WHO, the prevalence of T2DM in Pakistan for the year 2000 was 5.2 million, and for 2030, it is estimated to be 13.8 million. One-fourth of the population of Pakistan can be categorized as overweight or obese with the use of Indo-Asian-specific BMI cutoff values [4]. Developing or transitional economies are experiencing globalization of food markets, growing fast-food chains, and a rising trend of street vendors providing food items at affordable prices [5]. This is also the case for Pakistan, especially in urban areas, making this population susceptible to obesity and its associated complications.

Obesity and T2DM together are associated with significant morbidity and mortality due to cardiovascular disease [6]. Numerous measurements of obesity, with BMI, waist circumference, waist-to-hip ratio, and waist-to-stature ratio, are associated with the risk of T2DM [7]. In obese patients with T2DM, weight loss has a positive impact on the control of hyperglycemia [8]. Patient enthusiasm and knowledge are important factors in the management of both obesity and T2DM, and it is therefore important to evaluate the behavior and practices of T2DM patients with regard to obesity and related disorders.

There is a paucity of literature on this topic from developing countries such as Pakistan. The aim of this study is to evaluate the behavior and practices of type 2 diabetic (T2D) patients regarding obesity in Karachi, Pakistan.

## Materials and methods

A cross-sectional, population-based study was conducted in two tertiary care hospitals of Karachi (Civil Hospital and Pakistan Steel Hospital) during the period of December to February 2020 after getting approval from the Institutional Review Board of Dow University of Health Sciences. The sample population consisted of T2DM patients selected through convenience sampling. A sample size of 450 was taken through openepi.com with a 97% confidence level. Out of this, 417 completed the questionnaire fully, yielding a co-operation rate of 92.9%. Only residents of Karachi who were suffering from T2DM were included in this study. Patients with type 1 DM and gestational DM were excluded.

A structured questionnaire was used to carry out data collection. The questionnaire was available in both English and Urdu (the national language of Pakistan) to reduce linguistic barriers. Before data collection was started, the questionnaire was reviewed for relevance and completeness by two physicians independently, and a pilot study was also conducted and relevant changes were made. The questionnaire was divided into four sections containing 29 questions. The first section of the questionnaire consisted of sociodemographic information such as age, gender, weight, height, and level of education, as well as personal and family history of present medical illness. The second section dealt with comprehension regarding obesity, ideal body weight, BMI thresholds, and risk factors/causes of obesity. The third section assessed the behavior of the participants concerning obesity with coexisting T2DM and their willingness to lose weight, make dietary changes, and adopt exercise. The final section inquired about their weight loss regime (if any), dietary habits, and practices such as measurement of weight, blood pressure, and blood glucose level. Only recent and relevant information was asked in the questionnaire in order to minimize recall bias.

Verbal or written consent was taken from all participants after assuring them of complete confidentiality. The study investigators verbally interviewed those participants who were illiterate and completed the questionnaire on their behalf based on their oral responses. Interviewer bias was reduced here by training all interviewers to approach and interact with participants in a neutral and non-judgmental manner prior to carrying out data collection. No imputation method was used to account for missing data; incomplete questionnaires were excluded from the analysis.

T2DM was defined by the WHO definition, i.e., maintained fasting blood glucose of ≥7.0 mmol/L [9]. Respondents were categorized as obese (BMI ≥ 30) or non-obese (BMI < 30) per the WHO criteria. Data were analyzed using Statistical Package for the Social Sciences (SPSS) Version 24.0 (IBM Corp., Armonk, NY, USA).

## Results

Of the 417 T2D patients who participated in this study, 156 (37.4%) were obese. A majority of participants had a family history of either hypertension (n=262 [62.8%]) or T2DM (n=246 [60.0%]). The baseline characteristics of the study population are summarized in Table 1.

**Table 1:** Baseline characteristics of the study population

	Male	Female
Gender	200 (49.5%)	217 (50.5%)
Age		
<30 years	10	14
31-50 years	72	94
51-60 years	70	50
>60 years	48	55
Level of education		
No formal education	102	92
<12th grade (secondary school)	33	44
Graduate	59	49
Post-graduate	9	22

More than half the participants in our sample did not know the difference between obese and overweight (n=220 [52.8%]). Of the 265 participants who knew what their ideal body weight should be 221 (83.4%) knew how to measure it. High-calorie intake and lack of exercise were considered to be causes of obesity by a majority (n=264 [63.3%] and n=260 [62.4%], respectively) (Table 2).

**Table 2:** T2DM patients regarding obesity T2DM, type 2 diabetes mellitus

	Non-obese (n=261)	Obese (n=156)
Do you know the difference between obesity and overweight?		
Yes	140 (53.6%)	80 (51.3%)
Do you know the normal blood glucose level?		
Yes	174 (66.7%)	91 (58.3%)
Do you know the ideal body weight? And how to measure it?		
Yes/Yes	102 (39.1%)	66 (42.3%)
Yes/No	59 (22.6%)	38 (24.4%)
No/Yes	29 (11.1%)	24 (15.5%)
No/No	71 (27.2%)	28 (17.9%)
What do you think are the causes of obesity?		
Slow metabolism	90 (34.5%)	73 (46.8%)
High-calorie intake	167 (64.0%)	97 (62.2%)
Lack of exercise	163 (62.5%)	97 (62.2%)
Family history	81 (31.0%)	47 (3.1%)
No specific reasons	22 (8.4%)	11 (7.1%)
None of the above	9 (3.4%)	3 (1.9%)
What do you think are the risk factors for development of obesity?		
Diabetes mellitus	119 (45.6%)	97 (62.2%)
Cardiovascular diseases	88 (33.7%)	99 (63.5%)
Hypertension	98 (37.5%)	55 (35.3%)
High cholesterol	150 (57.5%)	93 (59.6%)
Joint pains/arthritis	95 (36.4%)	65 (41.7%)
None of the above	20 (7.7%)	10 (6.4%)

It was found that more obese participants were willing to reduce their weight (n=86 [55.1%]) than non-obese participants (n=126 [48.3%]) (Table 3). Among those who were willing to lose weight, this was mostly due to a wish to avoid further complications of obesity (n=155 [73.1%]) and also due to peer/family pressures (n=124 [58.5%]). Moreover, more obese (n=68 [43.6%]) than non-obese participants (n=87 [33.3%]) were willing to consult a doctor to help them reduce weight.

**Table 3:** Behavior of obese and non-obese T2DM patients regarding obesity T2DM, type 2 diabetes mellitus

	Non-obese (n=261)	Obese (n=156)
Are you willing to reduce your weight?		
Yes	126 (48.3%)	86 (55.1%)
If yes, then kindly select one or more of the following:		
Due to peer/family pressure	55 (21.1%)	69 (44.2%)
Avoid further complications	80 (30.7%)	75 (48.1%)
To do daily chores more efficiently	60 (23.0%)	48 (30.8%)
To fit in better in the society	18 (6.9%)	29 (18.6%)
Are you willing to consult a doctor to reduce your weight?		
Yes	87 (33.3%)	68 (43.6%)
Do you think overweight people should try to lose their weight?		
Yes	213 (81.6%)	121 (77.6%)
How important do you think it is for an obese person with diabetes to lose weight?		
Very Important	202 (77.4%)	134 (85.9%)
No need to lose weight	30 (11.5%)	14 (9.0%)
Diabetes and obesity are not related	26 (10.0%)	8 (5.1%)
Do you think having proper knowledge of your condition and treatment can help you reach a controllable state?		
Yes	192 (73.6%)	99 (63.5%)

**Figure 1:**
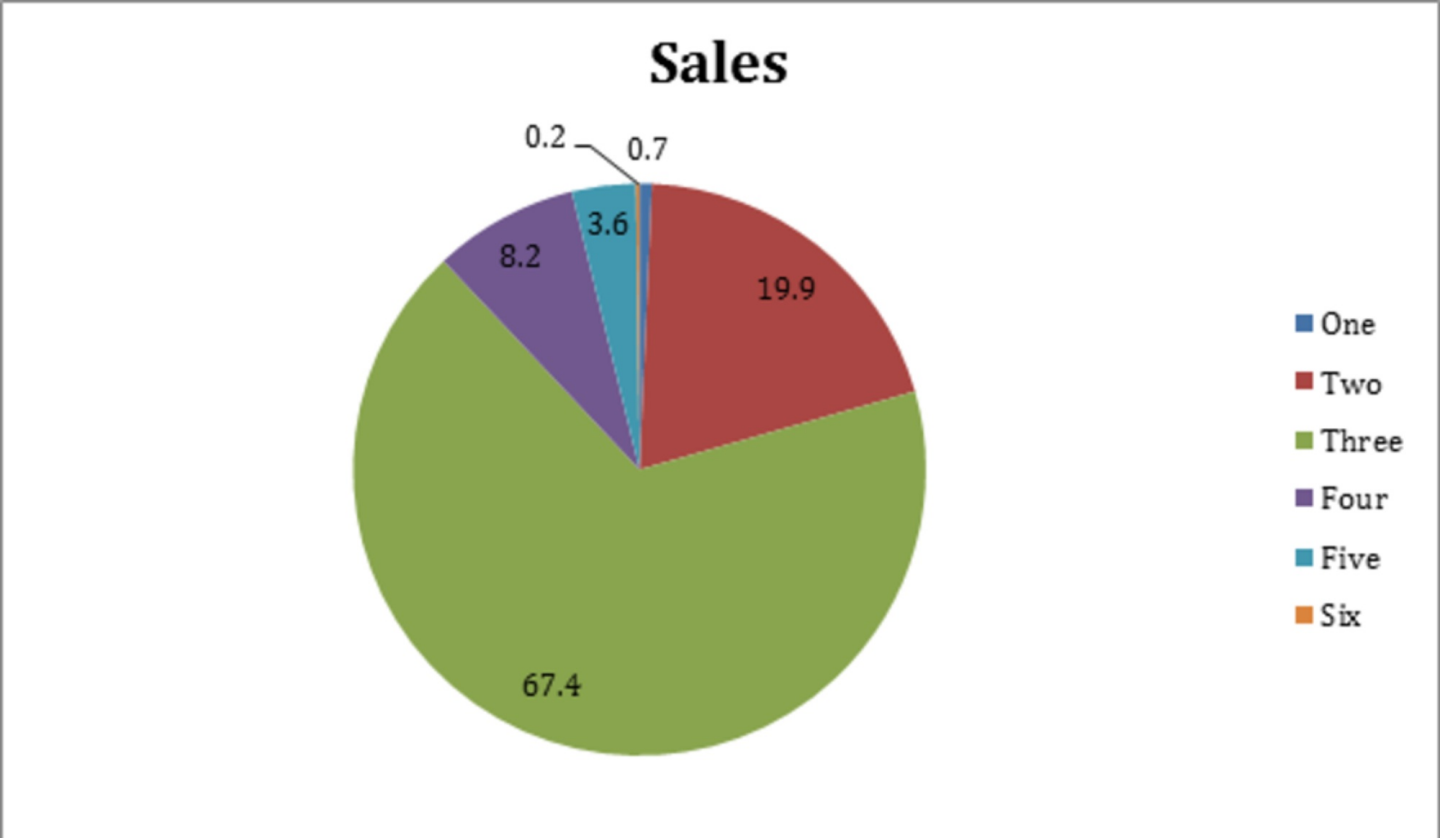
Number of meals per day taken by study participants

**Figure 2:**
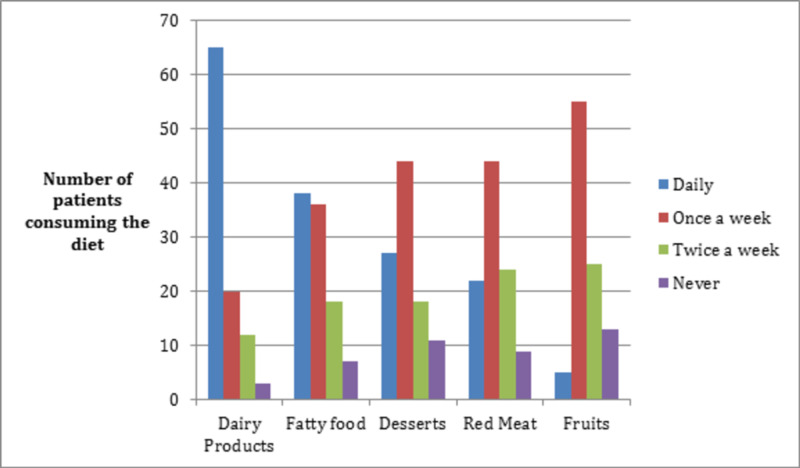
Diet composition of study participants

A greater percentage of obese (n=72 [46.2%]) than non-obese patients (n=57 [21.8%]) reported multiple previous attempts at losing weight. Participants had adopted various strategies to reduce weight, of which increasing exercise (n=242 [85.8%]) and healthy eating (n=162 [57.4%]) were most popular. Eating habits of patients, i.e., number of meals per day and diet composition, are shown in Figures 1, 2, respectively.

A greater proportion of obese participants exercised daily (n=71 [45.5%]) or had followed a special diet (n=65 [41.7%]) as compared to non-obese ones. Tables 4, 5 show the self-care practices of T2DM patients regarding obesity.

**Table 4:** Practices of obese and non-obese T2DM patients regarding obesity T2DM, type 2 diabetes mellitus

	Non-obese (n=261)	Obese (n=156)
Have you ever tried to lose weight in the past?		
Yes, many times	57 (21.8%)	72 (46.2%)
Yes, occasionally	98 (37.5%)	55 (35.3%)
Never tried	106 (40.6%)	29 (17.6%)
If yes, which of the following options have you followed to reduce your weight?		
Exercise	137 (52.5%)	105 (67.3%)
Weight-reducing medication	31 (11.9%)	32 (20.5%)
Avoiding meals	69 (26.4%)	49 (31.4%)
Eating healthy/reducing calorie intake	100 (38.3%)	62 (39.7%)
If you said yes to exercise, how often do you exercise?		
Daily	85 (32.6%)	71 (45.5%)
Few times a week	39 (15.0%)	27 (17.3%)
Few times a month	13 (4.9%)	7 (4.5%)
Have you ever been on a diet in an attempt to lose weight?		
Yes	91 (34.9%)	65 (41.7%)

**Table 5:** Self-monitoring practices of obese and non-obese T2DM patients T2DM, type 2 diabetes mellitus

	Non-obese (n=261)	Obese (n=156)
How often do you check your blood pressure?		
Daily	28 (10.7%)	24 (15.4%)
Alternate days	40 (15.3%)	21 (13.5%)
Twice a week	49 (18.8%)	41 (26.3%)
Once a week	71 (27.2%)	29 (18.6%)
Undocumented	63 (24.1)	41 (26.3%)
How often do you check your blood glucose level?		
Daily	39 (14.9%)	25 (16.0%)
Alternate days	41 (15.7%)	31 (19.9%)
Twice a week	49 (18.8%)	41 (26.3%)
Once a week	71 (27.2%)	29 (18.6%)
Undocumented	61 (24.4%)	30 (19.2%)
How often do you check your weight?		
Daily	23 (8.8%)	34 (21.8%)
Alternate days	20 (7.7%)	4 (2.6%)
Twice a week	24 (9.2%)	25 (16.0%)
Once a week	55 (21.1%)	32 (20.5%)
Undocumented	139 (55.6%)	61 (39.1%)

## Discussion

Obesity among T2DM patients is an alarming issue rising steadily worldwide and needs to be promptly addressed with efficient measures to reduce its economic burden [10]. A study carried out recently showed that being overweight also increases the risk of having cardiovascular and coronary diseases in a diabetic patient [11]. To deal with this issue, there is a need to educate people with T2DM about obesity and body weight reducing measures as patient education influences their health practices [12]. In our study, the majority of diabetics had a family history of obesity (129 [30.9%]) and hyper-cholesterolemia (134 [32.2%]) as proven by many types of researches that the fat-mass gene has a strong association with diabetes [13].

In this study, a considerable proportion of participants (n=220 [52.8%]) were unaware of the difference between overweight and obese or about how body weight is measured. This could be since in Pakistan, not every household has a weighing instrument as it is considered an unimportant resource to have, and gyms and places where they are available are unfortunately less popular. This is also reflected in the lack of consistency in weight self-monitoring practices among our participants. Many patients in our study were not regularly monitoring their blood pressure, weight, or blood sugar levels, underscoring the importance of the routine availability of appropriate instruments. Having a weighing machine is as important as having a glucometer in every household with T2DM patients since obesity can increase the risk of developing T2DM complications. Furthermore, clinicians should perceive the need for guidance, medication referral, and advice for weight loss regimens to be able to treat diabetes in their obese patients [14].

The lack of knowledge regarding ideal body weight in this study was surprisingly more profound as compared with a study carried out on newly diagnosed patients in Ghana. However, a satisfactory understanding of weight measurement techniques was found in patients from Ghana [15]. The majority of our patients knew that increased calorie intake (264 [63.3%]) and lack of exercise (260 [62.4%]) were risk factors for obesity, as also shown by studies carried out in Bangladesh [12], Ghana [15], and South Africa [16].

Obese T2DM patients were more willing to reduce weight as opposed to non-obese patients and were motivated by both health and social reasons. This could be due to health-related complications already faced by these patients, and a wish to fit in a society where body-shaming is highly prevalent [17]. Unfortunately, only 43.6% (n=68) of obese T2DM patients in our study shared a positive attitude toward consulting their physician for weight-loss counseling, making it imperative for physicians to initiate the discussion about the impact of obesity on the general health of T2DM patients [18].

More obese patients were seen to monitor their blood glucose (157 [37.6%]) and blood pressure (156 [37.4%]) levels daily as compared to non-obese ones, possibly due to an understanding that they were at higher risk of heart disease and hypertension than non-obese T2DM patients. In Pakistan, three meals are typically consumed in a day, and fried oily food and dairy products are part of the normal diet regimen. This dietary pattern is consistent with our study findings and a previous study conducted in Pakistan in Aga Khan Hospital Karachi [19]. The popular trend of only “eating out” in Pakistan further makes it challenging for T2DM patients to avoid high-calorie foods [20].

According to a study carried out in Malaysia [21], most diabetic patients showed a positive attitude toward making lifestyle changes; however, they failed to implement them. Another study conducted in Pakistan similarly found that although T2DM patients had profound knowledge about their disease, they failed to apply their knowledge to change their behavior [22]. This highlights the need for public health initiatives that go beyond increasing health awareness and motivate and guide patients on how to make sustainable changes to their diet and physical activity routine.

There have been many successfully executed lifestyle modification programs, such as the U.S. Diabetes Prevention Program (US DPP) and Finnish Diabetes Prevention Program, which provide their participants with weekly guidelines on ways to reduce weight and eat healthily [13,23-25]. Based on these models, similar campaigns can and should be developed in Pakistan too given its current burden of these two chronic illnesses.

Besides large-scale efforts to increase public awareness, physicians also have a critical role in addressing barriers to weight loss among T2DM patients and the need to provide patients with pragmatic guidelines and support on this issue. Physicians should further make appropriate referrals to nutritionists, physical therapists, and bariatric surgery, as needed.

## Conclusions

This study might aid in the application of diabetic education programs, giving more stress on elder and younger groups of patients and fortify the patients to have more connection with concerning physicians so that complications at the early stage of the disease could be prevented.
